# Expanding interpretability through complexity reduction in machine learning‐based modelling of cardiovascular disease: A myocardial perfusion imaging PET/CT prognostic study

**DOI:** 10.1111/eci.14391

**Published:** 2025-04-07

**Authors:** Eero Lehtonen, Jarmo Teuho, Monire Vatandoust, Juhani Knuuti, Remco J. J. Knol, Friso M. van der Zant, Luis Eduardo Juárez‐Orozco, Riku Klén

**Affiliations:** ^1^ Turku PET Centre Turku University Hospital and University of Turku Turku Finland; ^2^ Cardiac Imaging Division Alkmaar, Department of Nuclear Medicine Northwest Clinics Alkmaar The Netherlands; ^3^ Department of Cardiology, Division Heart & Lungs University Medical Center Utrecht, Utrecht University Utrecht The Netherlands

**Keywords:** data analytics, interpretability, machine learning, myocardial perfusion imaging, positron emission tomography

## Abstract

**Background:**

Machine learning‐based analysis can be used in myocardial perfusion imaging data to improve risk stratification and the prediction of major adverse cardiovascular events for patients with suspected or established coronary artery disease. We present a new machine learning approach for the identification of patients who develop major adverse cardiovascular events. The new method is robust against the deleterious effect of outliers in the training set stratification and training process.

**Methods:**

The proposed sum‐of‐sigmoids model is obtained by averaging the contributions of various input variables in an ensemble of XGBoost models. To illustrate its performance, we have applied it to predict major adverse cardiovascular events from advanced imaging data extracted from rest and adenosine stress ^13^N‐ammonia positron emission tomography myocardial perfusion imaging polar maps. There were 1185 individual studies performed, and the event occurrence was tracked over a follow‐up period of 2 years.

**Results:**

The sum‐of‐sigmoids model achieved a prediction accuracy of .83 on the test set, matching the performance of significantly more complex and less interpretable models (whose accuracies were .83–.84).

**Conclusion:**

The sum‐of‐sigmoids model is interpretable and simple, while achieving similar prediction accuracy to significantly more complex machine learning models in the considered prediction task. It should be suitable for applications such as automated clinical risk stratification, where clear and explicit justification of the classification procedure is highly pertinent.

## Graphical abstract

In this work we propose the *sum‐of‐sigmoids* model, which is obtained by averaging the contributions of input variables in an ensemble of XGBoost models. The proposed model is simple and interpretable, and yet it achieves similar accuracy scores to significantly more complex machine learning models in predicting major cardiovascular events from ^13^N‐ammonia PET myocardial perfusion polar maps. An additional advantage of the proposed model is its robustness against outlier effects in training set stratification and the training process.

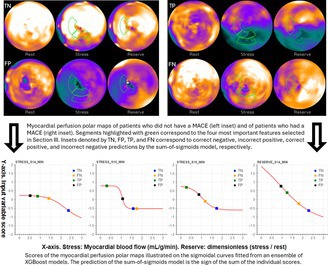

## 1 INTRODUCTION

Chronic coronary syndromes represent the most common cause of death globally.^1^, ^2^ Despite substantial developments in advanced cardiac imaging, accurate risk stratification and therefore prediction of acute events (major adverse cardiovascular events (MACE)) derived from myocardial ischemia, thromboembolism, heart decompensation and cardiac death remains a major challenge for clinicians and an unmet need in cardiology.^1^

Positron emission tomography (PET) myocardial perfusion imaging (MPI) enables quantitative assessment of myocardial blood flow (MBF) and flow reserve (MFR), thus allowing to detect functionally significant coronary artery stenoses and myocardial ischemia.^3^ Perfusion quantification and the extent of ischemic burden have demonstrated predictive value for the development of MACE.^4^, ^5^, ^6^, ^7^, ^8^ Notably, MPI studies deliver a vast number of qualitative and quantitative individual variables, which cannot be easily managed by the human operator. Therefore, these studies are rendered in polar maps for clinical interpretation with vessel territory segmentation to facilitate their visualization by human operators.

Machine learning‐based analysis can be used in myocardial perfusion imaging data to improve risk stratification and MACE prediction for patients with suspected or established coronary artery disease (CAD), as previously demonstrated, for example, in.^9^, ^10^ In^9^ a deep learning model using PET myocardial perfusion polar maps was able to outperform clinical, functional, and quantitative flow variables in identifying patients who develop MACE. A major drawback of such an approach is that interpretability is often restricted by the complexity of the model and the number of non‐linear dependencies at play. Recently^11^ machine learning through boosted ensembles (XGBoost^12^) was applied for the identification of patients that are likely to benefit from subsequent PET MPI based on clinical and CCTA variables. Although effective, explainability may also be hampered by the complexity of the modelling.

Therefore, the present work describes the exploration of new machine learning‐based approach for the identification of patients who develop MACEs from the analysis of rest, stress, and reserve myocardial perfusion polar maps obtained from ^13^N‐ammonia PET/CT MPI studies. The proposed sum‐of‐sigmoids model is highly interpretable, while yielding similar prediction scores as more complex baseline models (in this work we compare the sum‐of‐sigmoids model to a convolutional neural network, a logistic regression model, and an XGBoost model). The decision rule of the sum‐of‐sigmoids model is explicitly obtained from a small set of sigmoid curves of perfusion statistics, and therefore is far more interpretable than just a feature importance graph of many input variables. This contrasts with the considered baseline models: a convolutional neural network is a black box with thousands or millions of learned parameters. Similarly, the considered XGBoost baseline model is based on a large set of nested decision rules, while the considered logistic regression is allowed to use all the considered variables (and not just a small subset as the presented sum‐of‐sigmoids model). The proposed methodology seems to work notably well for the considered objective, and additionally, is general enough to be considered for other classification applications. Its interpretability (scoring is based on sigmoid curves of perfusion statistics) and simplicity (prediction is based on a sum of scores of a small number of features) supports its potential for the improvement of automated risk stratification. It could also provide insights into which perfusion statistics are most relevant in identifying patients at risk of MACE.

## 2 METHODS

### 2.1 Data acquisition and preprocessing

Data from 1185 patients was retrospectively collected and analysed from the population referred to quantitative ^13^N‐ammonia PET myocardial perfusion imaging due to suspected myocardial ischemia between 2015 and 2017 at the department of nuclear medicine of the Northwest Clinics, Alkmaar, the Netherlands. Patients with documented CAD either as prior myocardial infarction (MI) or revascularization (percutaneous coronary intervention or coronary artery bypass graft surgery), were excluded from the study. After the removal of invalid data entries, polar maps from 1079 patients were retained for further analysis. Details on the study population, data collection, and the acquisition‐reconstruction protocol are presented in Appendix S1 and in^13^ and^14^, while the quantification of the myocardial blood flow (MBF) and flow reserve (MFR) in the MPI polar maps is presented in detail in^15^. For each patient it was recorded whether or not they had MACE during a follow‐up period of 2 years. In this work we aim to predict the value of this binary variable based on numerical features obtained from the PET perfusion study.

2.2

For each patient, the stress, rest, and reserve perfusion polar maps were automatically segmented to the standard 17 segments^16^ using a Python script. For each segment, the following 13 statistics were computed: the mean, the standard deviation, the minimum value, the maximum value and the 10th, 20th, … and 90th percentiles. To summarize, for each patient

k=3·17·13=663.

numerical features were computed from the three perfusion polar maps.

This dataset of polar map numerical features was randomly divided into a training set and a test set with 3:1 ratio. As a result, the training set contains data corresponding to *N*_1_ = 809 patients, and the test set contains data corresponding to *N*_2_ = 270 patients. Notice that the 13 statistics are computed independently for each segment, and therefore there is no risk of information leakage in the train‐test split.

Appropriate data division for the numerical features was verified using false discovery rate‐corrected Kruskal's test with *p* = .05. For the binary outcome variable (MACE) we tested that ratios

$$r_{1}=\frac{\#\left(\text{patients without MACE in training}\;\mathrm{set}\right)/N_{1}}{\#\left(\text{patients without MACE in test}\;\mathrm{set}\right)/N_{2}}$$

and

$$r_{2}=\frac{\#\left(\text{patients with MACE in training}\;\mathrm{set}\right)/N_{1}}{\#\left(\text{patients with MACE in test}\;\mathrm{set}\right)/N_{2}}$$

satisfy

$$\frac{1}{1.1}<r_{i}<1.1;i=1,2.$$

Of the 809 patients in the training dataset, 102 had a MACE during the follow‐up period. Due to this significant imbalance in outcomes, the following simple stratification approach was used when training the XGBoost models: negative cases were discarded at random to obtain a training set with approximately 40% positive (a MACE was recorded) and 60% negative (no MACE was recorded) cases. This ratio was selected as it is close to uniform distribution, but yields a slightly larger training sets, which is beneficial in training the models. For each XGBoost model considered in this work, an independent and random stratification was performed on the training dataset.

**FIGURE 1:**
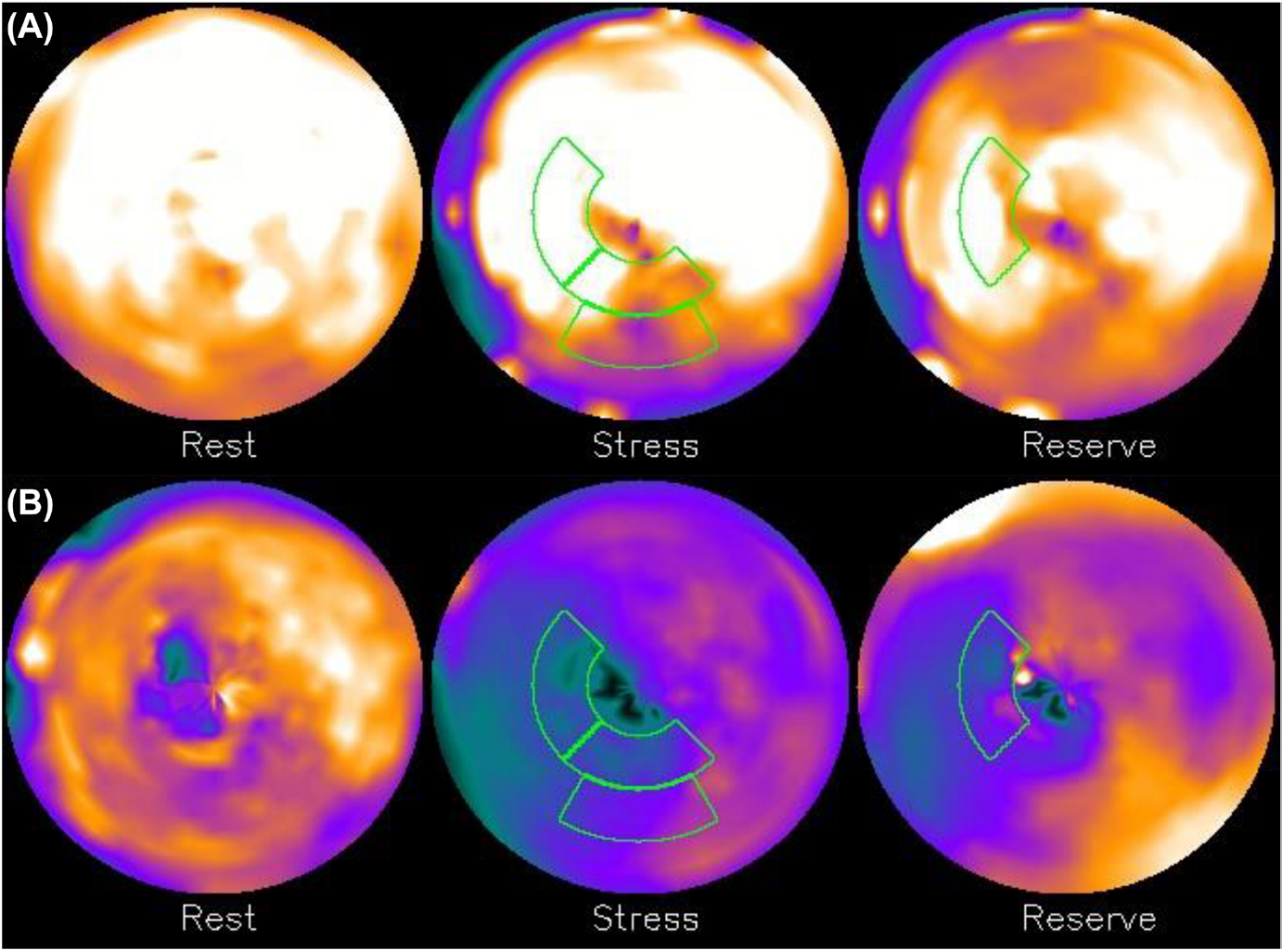
Myocardial perfusion polar maps of two patients who did not have a MACE during the two‐year follow‐up period. Segments highlighted with green correspond to the four most important features selected in Section II‐E. Inset (A): Correct negative prediction by the sum‐of‐sigmoids model described in Section III, inset (B): Incorrect positive prediction by the sum‐of‐sigmoids model.

**FIGURE 2:**
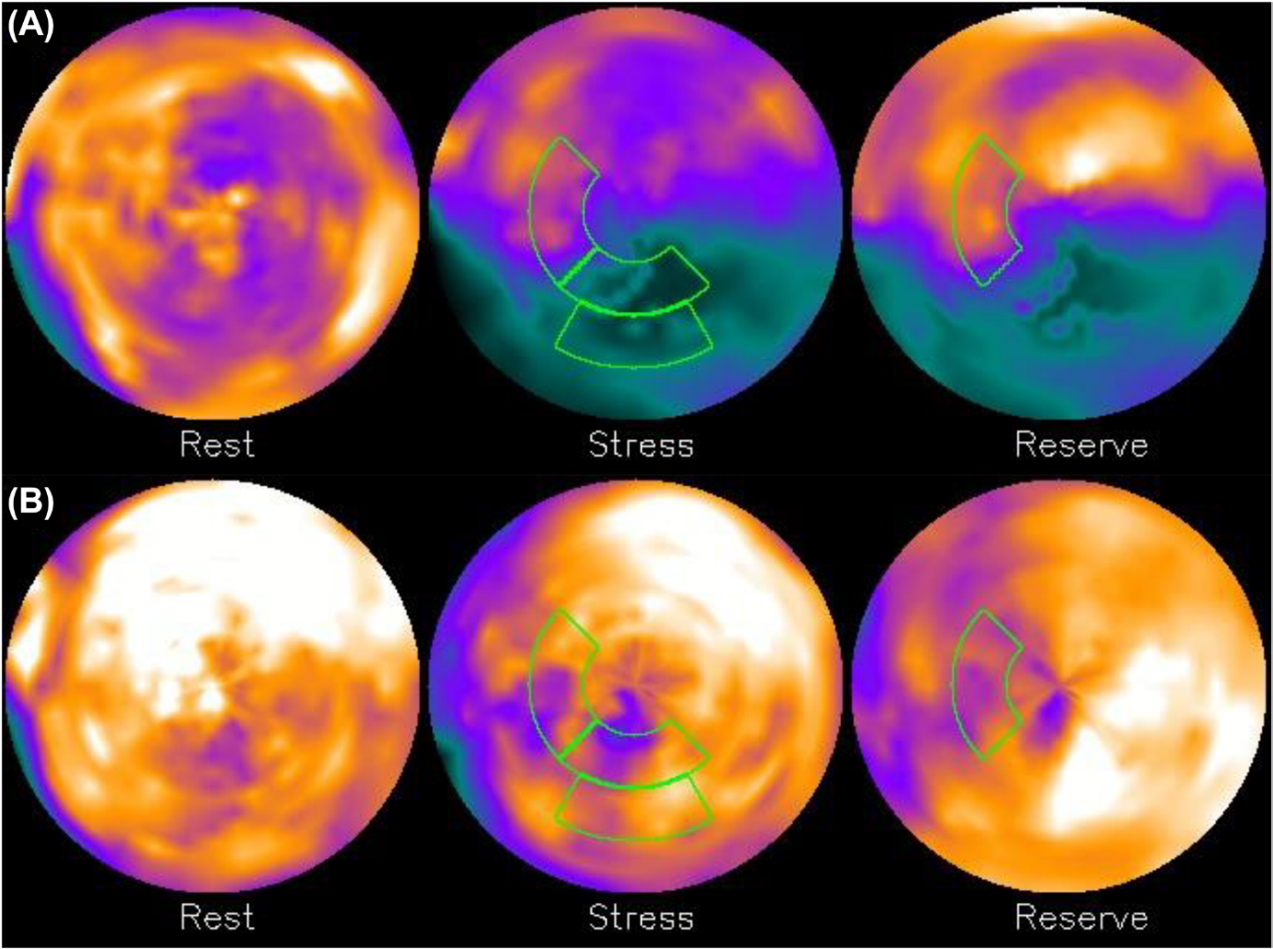
Myocardial perfusion polar maps of two patients who had a MACE during the two‐year follow‐up period. Segments highlighted with green correspond to the four most important features selected in Section II‐E. Inset (A): Correct positive prediction by the sum‐of‐sigmoids model described in Section III, inset (B): Incorrect negative prediction by the sum‐of‐sigmoids model.

Examples of myocardial perfusion polar maps found in the considered dataset are depicted in Figures 1 and 2. The segments highlighted with green correspond to the most important features found by the feature selection process described in Section II‐E. As can be seen, prediction of MACE based solely on perfusion maps is not a trivial task; a myocardial perfusion polar map with relatively high perfusion values—such as the one depicted in Figure 2B—may still correspond to a patient who had a MACE, while a polar map with relatively low perfusion values may correspond to a patient who did not have a MACE (an example of this is illustrated in Figure 1B).

### 2.3 Machine learning models

In this work we considered the following machine learning models: Convolutional Neural Network (CNN), logistic regression, eXtreme Gradient Boosting (XGBoost), and the new sum‐of‐sigmoids model. CNN, logistic regression and XGBoost baseline models were used to obtain classification scores for the evaluation of the sum‐of‐sigmoids model.

As the first baseline model, we trained a CNN model^17^ previously used for ischemia detection in ^15^O‐H_2_O perfusion studies (using only reserve polar maps) using the ^13^N‐ammonia reserve polar maps. Classification results of the CNN model on the ^13^N‐ammonia test set can be seen in Table 1.

**TABLE 1:** Baseline CNN model. Classification statistics in the test set: #TN = 202, #FP = 32, #FN = 11, #TP = 25, where #TN, #FP, #FN, and #TP denote the numbers of true negatives, false positives, false negatives, and true positives, respectively.

	Precision	Recall	f1‐score
Patients without a MACE	.95	.86	.90
Patients with a MACE	.44	.69	.54
Accuracy			.84
Weighted averages	.88	.84	.85

As the second baseline model, an XGBoost model was trained on a stratified training set (using all of the 663 numerical features), with the following hyperparameters:

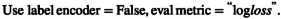




Classification results for this model are presented in Table 2. The performance is quite similar to the baseline CNN model; the main difference seems to be that the baseline XGBoost model yields less false negatives and more false positives than the baseline CNN model. As the third baseline model we trained a logistic regression model with LASSO (based on L1 regularization) on a stratified training set using all the 663 numerical features. The classification results are presented in Table 3. As presented in Table 5, the logistic regression model’s performance is very similar to the baseline XGBoost model’s performance.

**TABLE 2:** Baseline XGBoost model which uses all the 663 numerical features. Classification statistics in the test set: #TN = 191, #FP = 43, #FN = 4, #TP = 32.

	Precision	Recall	f1‐score
Patients without a MACE	.98	.82	.89
Patients with a MACE	.43	.89	.58
Accuracy			.83
Weighted averages	.91	.83	.85

**TABLE 3:** Logistic regression with LASSO. Classification statistics in the test set: #TN = 192, #FP = 42, #FN = 5, #TP = 31.

	Precision	Recall	f1‐score
Patients without a MACE	.97	.82	.89
Patients with a MACE	.42	.86	.57
Accuracy			.83
Weighted averages	.90	.83	.85

### 2.4 Feature selection

Next, 100 XGBoost models with the same hyperparameters as above and with the maximum depth of one were trained on independently stratified training sets. The maximum depth of one means that the XGBoost models consist of independent tests, each of which considers only a single input variable. Each test obtains a gain value (output of the function call get booster().get score(importance type=’gain’) in the XGBoost library) associated with the tested input variable, which represents the importance of that test and variable in the overall classification task. By summing up all gains of a specific input variables over all the trained XGBoost models, we can sort the input variables in order of decreasing sum gain. The sorted top 20 input variables obtained in this manner (listed in decreasing importance) are STRESS_S14_MIN, STRESS_S10_MIN, STRESS_S15_MIN, RESERVE_S14_MIN, STRESS_S16_MIN, STRESS_S17_P20, STRESS_S10_P10, STRESS_S15_P10, RESERVE_S4_P10, RESERVE_S9_P10, RESERVE_S9_SD, RESERVE_S14_SD, RESERVE_S17_MIN, RESERVE_S9_MIN, STRESS_S17_MIN, RESERVE_S14_P10, STRESS_S14_SD, RESERVE_S4_P30, RESERVE_S10_SD and RESERVE_S17_P20.

Here S refers to the segment number (for example, S14 refers to segment number 14), MIN refers to the minimum value, SD to the standard deviation and P10 to the 10th percentile (similar notation is used for other percentiles). As can be seen, all the variables on this list are either from stress or the reserve polar maps. The first rest polar map‐based variable was found to be the 36th most important. We note that the 20 most important input variables are all either minimum values, low percentiles (from 10th to 30th percentiles) or standard deviations.

### 2.5 Sum‐of‐sigmoids model

The sum‐of‐sigmoid model is derived from an ensemble of XGBoost models, whose maximum depth is one. Let us denote the number of the XGBoost models in the ensemble by *N*; in the results presented in this work *N* = 100. Each of the XGBoost models comprises of a set of tests (a single test considers one of the input variables) of the type

- Is the considered input variable larger than a threshold *θ*? If it is, add *v*_1_ to the overall score; otherwise, add *v*_2_.

For each of the input variables, we compute an *input variable score curve* by computing the average effect of the tests corresponding to that variable over the XGBoost models. The process of computing the input variables score curve *s*(*x*) is defined in Algorithm 1.

Algorithm 1 FOR EACH INPUT VARIABLE *i*: SET *s*_*i*_(*x*) = 0 FOR EACH XGBOOST MODEL IN THE ENSEMBLE: FOR EACH TEST OF INPUT VARIABLE *i*, SET: *s*_*i*_(*x*) += (*v*_1_ · *H*(*x* − *θ*)+ *v*_2_ · (1 − *H*(*x* − *θ*)))/*N*, WHERE *θ*, *v*_1_, *v*_2_ ARE THE TEST PARAMETERS. RETURN *s*_*i*_(*x*)

Here H(*x*) is the Heaviside step function:

$$H\left(x\right)=\left\{\begin{array}{ll} 1, & x\geq 0\\ 0, & \text{otherwise}. \end{array}\right.$$

Next, each input variable score curve $s_{i}\left(x\right)$ is approximated by curve fitting a sigmoid function of the form

$$f_{i}\left(x\right)=A_{i}\left(\frac{1}{1+\exp\left(k_{i}\left(x-t_{i}\right)\right)}-m_{i}\right),$$

where $A_{i}$, $t_{i}$, $k_{i}$ and $m_{i}$ are the fitting parameters.

Sigmoids can be used to approximate both linear functions (within finite ranges) as well as functions which saturate either to some minimum or some maximum values, so long as the functions are monotonous. We expect that myocardial blood flow values should affect the prediction of MACE monotonously (for example, the larger the value, the less likely it is to have a MACE); the evaluation of this model will show if our intuitive expectation is well justified.

Finally, the sum‐of‐sigmoid model proposed in this paper is a binary predictor, where the sigmoid functions corresponding to each of the input variables are summed together and passed through the Heaviside step function:

$$\hat{y}=H\left(\sum_{i=1}^{N}f_{i}\left(x_{i}\right)\right),$$

where $\hat{y}$ denotes the binary prediction (where $\hat{y}=0$ denotes the prediction of no MACE and $\hat{y}=1$ the prediction of a MACE).

## 3 RESULTS

Let us consider the four most important input variables STRESS_S14_MIN, STRESS_S10_MIN, STRESS_S15_MIN and RESERVE_S14_MIN.

**FIGURE 3:**
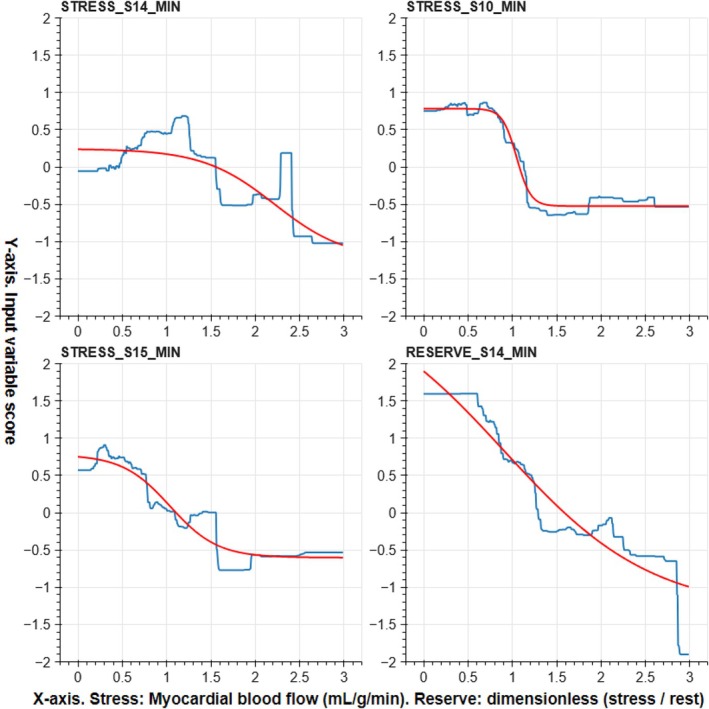
Input variable score curves for the four most important features in blue, and their sigmoid approximations in red.

Their input variable score curves and approximations by sigmoid functions are represented in Figure 3.

Let us denote these sigmoid approximations as *f*_*i*_(*x*_*i*_), where *i* = 1, …, 4. The variable *x*_*i*_ corresponds to the perfusion statistics (here, each variable is a minimum value of a myocardial blood flow statistic) in the considered polar map and segment, and the value *f*_*i*_(*x*) is a score which represents the average effect of that perfusion statistics to the overall decision made by the ensemble of the XGBoost models. Now the final sum‐of‐sigmoids model can be written simply as

$$\hat{y}=H\left(\sum_{i=1}^{4}f_{i}\left(x_{i}\right)\right).$$

If the sum of the sigmoids is negative, no MACE is predicted, and if it is positive, a MACE is predicted. Notice that this model is no longer an XGBoost model, although several XGBoost models were used to define it. Classification results for this model are presented in Table 4. The results are similar to the ones obtained with the baseline models, but the sum‐of‐sigmoid model with four input features is clearly much easier to interpret than the baseline models.

**TABLE 4:** Sum‐of‐sigmoids model with four input features. Classification statistics in the test set: #TN = 196, #FP = 38, #FN = 7, #TP = 29.

	Precision	Recall	f1‐score
Patients without a MACE	.97	.84	.90
Patients with a MACE	.43	.81	.56
Accuracy			.83
Weighted averages	.89	.83	.85

**FIGURE 4:**
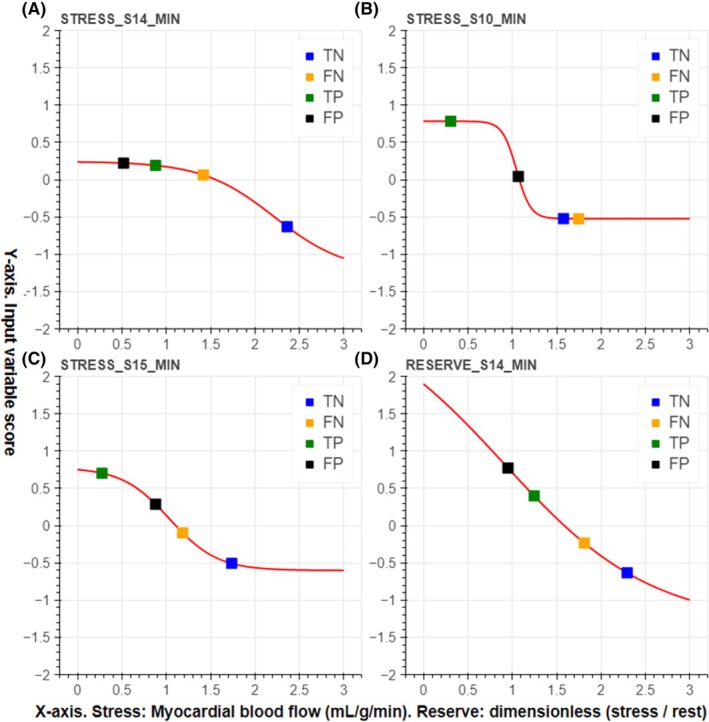
Scores of four different myocardial perfusion polar maps corresponding to Figure 1(TN corresponds to inset (a) and FP corresponds to inset (b)) and Figure 2 (TP corresponds to inset (a) and FN corresponds to inset (b)). In this figure, inset (A) illustrates the input variable score curve for the variable STRESS_S14_MIN, inset (B) illustrates the input variable score curve for STRESS_S10_MIN, inset (C) illustrates the input variable score curve for STRESS_S15_MIN, and inset (D) illustrates the input variable score curve for RESERVE_S14_MIN. The individual scores are presented by colored squares. Here, TN stands for the true negative (no MACE) case which was correctly predicted, FP stands for the false positive (no MACE) case which was incorrectly predicted, TP stands for the true positive (MACE) case, which was correctly predicted, and FN stands for the false negative (MACE) case which was incorrectly predicted.

Figure 4 illustrates input variable scores obtained from myocardial perfusion polar maps depicted in Figures 1 and 2. Four different cases are illustrated based on the prediction by the sum‐of‐sigmoids model: true negative (TN) having as input Figure 1A, false negative (FN) having as input Figure 2B, true positive (TP) having as input Figure 2A and false positive (FP) having as input Figure 1B. Here the sum of the four scores associated with the TN and FN cases is negative (no MACE is predicted), and the sum of the scores associated with the TP and FP cases is positive (a MACE is predicted).

## 4 DISCUSSION

The need for interpretable machine learning models for the analysis of myocardial perfusion maps has been widely recognized. In^18^ a radiomics approach was applied to myocardial retention images to identify patients with reduced global myocardial flow reserve. The main focus of^18^ was on the extraction and usefulness of the radiomic features—which have some correlation to the variables considered in this work—and only univariate logistic regression analysis was applied for classification. In contrast, our work concentrates on the development of a novel prediction method. Of note, the Smote‐XGBoost algorithm for predicting cardiac events from patient‐derived data (numeric and categorical variables, not restricted to medical imaging) has been previously presented in.^19^ Similarly to our work, the most important features are selected using the XGBoost gain value. However, their dataset was substantially different, and this reflected in the final prediction method (XGBoost was found to be the most effective prediction model out of many other machine learning methods). In our previous work,^20^ we found XGBoost to be an efficient method for predicting cardiac events using clinical variables, coronary computed tomography angiography and ^15^O‐H_2_O PET‐based myocardial perfusion values. An interesting topic for future work is to extend the current sum‐of‐sigmoids model to include also clinical variables as input variables.

Our approach is related to the surrogate models in machine learning.^21^ Surrogate models are models that can enhance interpretability, while having been trained using the outputs of more complex models. However, a significant difference is that the proposed sum‐of‐sigmoids model is not trained but constructed using curve‐fitting—where each input variable is fitted independently—from an ensemble of XGBoost models. This means that we did not need to apply any learning method such as gradient descent to our final sum‐of‐sigmoids model, which significantly simplifies the overall procedure.

Table 5 lists the prediction performance of the considered machine learning models. It can be seen that no one model performed the best in every considered classification performance category. The proposed sum‐of‐sigmoids model is a good middle ground approach that doesn't achieve the best performance value in any of the categories but also avoids the worst performance values. At the same time, it is the most easily explainable model together with the logistic regression model.

**TABLE 5:** Prediction accuracies and classification performance values for the considered machine learning models. BL XGBoost and Log. reg. with LASSO are baseline model which receives all the 663 numerical features as inputs.

Method	CNN	BL XGBoost	Log. reg. with LASSO	Sum‐of‐sigmoids
Accuracy	.84	.83	.83	.83
#TN	202	191	192	196
#FP	32	43	42	38
#FN	11	4	531	7
#TP	25	32	31	29

**FIGURE 5:**
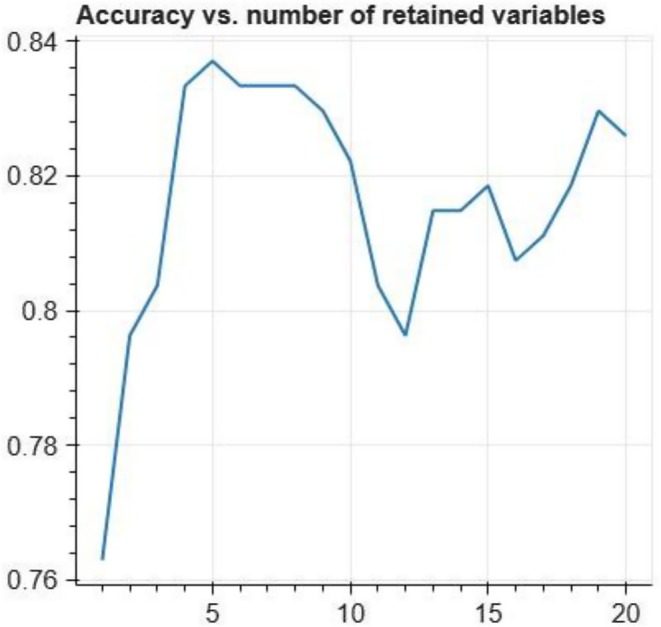
Classification accuracy of the sum‐of‐sigmoids models as the function of the number of input variables included in the final model.

As explained in Section II‐E, we selected the four most important numerical features as the variables of the sum‐of‐sigmoids model. Figure 5 shows how the classification accuracy depends on the number of input features in the final model. As can be seen, accuracy does not significantly improve when using more than four input variables with this data.

Interestingly, when adding the 11th input variable to the set of input variables of the model, the accuracy drops. One should note that although the ten other input variables are the ones used in the 10‐variable model, the underlying XGBoost models can be quite different, which also yields different input variable curves. In practice, we recommend plotting the accuracy versus number of parameters curve and to select the number of input variables from a ‘stable’ part on the curve. However, this nonmonotonicity of the accuracy curve should be investigated in more detail in future.

The range of the input variable score curve corresponding to RESERVE_S14_MIN is the largest of the four illustrated in Figure 3, although it was the fourth most important variable. It seems then that the sum gain of a variable, obtained from the XGBoost library, is not directly connected to the range of the input variable score curve. This naturally raises the question whether the sum gain represents the best criterion for selecting the input variables for the proposed sum‐of‐sigmoids model. This question, however, should be answered in future research.

As a practical note, in the case of many input features, one can discard sigmoid functions which have a small output range (smaller than some threshold) from the final model; this does not affect much the overall performance and may simplify the model significantly.

As can be seen, the logistic regression model (based on the 4 most important features) has similar performance as compared to the sum‐of‐sigmoids model. However, it should be noted that the sum‐of‐sigmoids model is less sensitive to the selection of the balanced training set as it is the result of 100 models. A more sophisticated linear model could be obtained, for example, by training separate linear models for each of the most important features and summing these models for the final prediction. Such a model would prove more immune to missing values in the inputs. Averaging weights and biases of several linear models trained with different stratified training sets could also yield a more robust model. Such improvements of the linear and logistic regression models are outside the scope of this work, and comparison of such models against the proposed sum‐of‐sigmoids model warrants further investigation.

The sum‐of‐sigmoids model presented in this work is based on an ensemble of XGBoost models (here, the ensemble consists of 100 XGBoost models), each of which is trained on an independently chosen stratified training set. The resulting model is not sensitive to any specific training set stratification or XGBoost model performance but is rather based on how the XGBoost models on average treat different input variables. The sigmoid input variable score curves show explicitly how different variables affect the final score (positive values steer the classification towards prediction of an event), and the ranges of different score curves determine how strongly different input variables affect the prediction. The final classification result can be easily visualized by highlighting the segments corresponding to the input variables and by showing the scores on the sigmoid curves as represented in Figures 1, 2 and 4. This makes the presented model easily interpretable, which in turn supports its potential for automated risk stratification, where clear and explicit justification of the classification procedure is highly pertinent.

An underlying assumption of the proposed method is that the prediction task can be performed with independent tests of input variables, as each XGBoost model in the ensemble has a unit depth. The PET MPI polar map‐based prediction task seems to satisfy this assumption, as the performance of the sum‐of‐sigmoids model matches that of more holistic and complex approaches, such as the convolutional neural network or the XGBoost model with depth of six. In the future, we will additionally investigate the applicability of this method to other datasets and applications, including PET studies with different radiotracers.

## 5 CONCLUSION

In this work we proposed a sum‐of‐sigmoids model, which is obtained by averaging the contributions of input variables in an ensemble of XGBoost models. The proposed model is simple and interpretable, and yet it achieves similar accuracy scores to significantly more complex machine learning models in predicting major cardiovascular events from ^13^N‐ammonia PET perfusion imaging polar maps. In addition to interpretability, an advantage of the proposed sum‐of‐sigmoids model is its robustness against outlier effects in training set stratification and the training process. We believe that the proposed model could be highly useful in automated clinical risk stratification, where clear and human‐understandable justification of the decision is of utmost importance. As this is the first work to propose and analyse the sum‐of‐sigmoids model, we have also identified several important directions for future research.

## AUTHOR CONTRIBUTIONS

E.L. invented the sum‐of‐sigmoids model, wrote codes regarding it, and wrote most of the manuscript. J.T. and M.V. performed the evaluation of the deep learning baseline model, wrote algorithms for data preprocessing and contributed to the writing of the manuscript. J.K. and R.K. oversaw the scientific work and contributed to the writing of the manuscript. L.J., R.K. and F.Z. contributed to the data collection, inception, analysis interpretation and report structuring in the present work.

## CONFLICT OF INTEREST STATEMENT

Dr. Knuuti received consultancy fees from GE Healthcare and Synektik and speaker fees from Bayer, Lundbeck, Boehringer‐Ingelheim, Pfizer and Siemens, outside of the submitted work. All other authors have reported that they have no relationships relevant to the contents of the paper to disclose.

## Supporting information


Appendix S1.

## Data Availability

The data that support the findings of this study are available from the corresponding author upon reasonable request.

## References

[1] Knuuti J , Wijns W , Saraste A , et al. 2019 ESC guidelines for the diagnosis and management of chronic coronary syndromes. Eur Heart J. 2020;41(3):407‐477. 31504439 10.1093/eurheartj/ehz425

[2] GBD 2015 Mortality and Causes of Death Collaborators . Global, regional, and national life expectancy, all‐cause mortality, and cause‐specific mortality for 249 causes of death, 1980–2015: a systematic analysis for the global burden of disease study 2015. Lancet. 2016;388(10053):1459‐1544. 27733281 10.1016/S0140-6736(16)31012-1 PMC5388903

[3] Nakazato R , Berman DS , Alexanderson A , Slomka P . Myocardial perfusion imaging with PET. Imaging Med. 2013;5(1):35‐46. 23671459 10.2217/iim.13.1 PMC3650901

[4] Juárez‐Orozco LE , Tio RA , Alexanderson E , et al. Quantitative myocardial perfusion evaluation with positron emission tomography and the risk of cardiovascular events in patients with coronary artery disease: a systematic review of prognostic studies. Eur Heart J Cardiovasc Imaging. 2018;19(10):1179‐1187. 29293983 10.1093/ehjci/jex331 PMC6148746

[5] Chen A , Wang H , Fan B , Xu Y , Chen W , Dai N . Prognostic value of normal positron emission tomography myocardial perfusion imaging in patients with known or suspected coronary artery disease: a meta‐analysis. Br J Radiol. 2017;90(1074):20160702. 28306335 10.1259/bjr.20160702 PMC5602171

[6] Dorbala S , Di Carli MF , Beanlands RS , et al. Prognostic value of stress myocardial perfusion positron emission tomography: results from a multicenter observational registry. J Am Coll Cardiol. 2013;61(2):176‐184. 23219297 10.1016/j.jacc.2012.09.043 PMC3549438

[7] Rauf M , Hansen KW , Galatius S , et al. Prognostic implications of myocardial perfusion imaging by 82‐rubidium positron emission tomography in male and female patients with angina and no perfusion defects. Eur Heart J Cardiovasc Imaging. 2023;24(2):212‐222. 36394344 10.1093/ehjci/jeac217

[8] Bom MJ , van Diemen PA , Driessen RS , et al. Prognostic value of [^15^O]H_2_O positron emission tomography‐derived global and regional myocardial perfusion. Eur Heart J Cardiovasc Imaging. 2020;21(7):777‐786. 31620792 10.1093/ehjci/jez258

[9] Juárez‐Orozco LE , Martinez‐Manzanera O , van der Zant FM , Knol RJJ , Knuuti J . Deep learning in quantitative PET myocardial perfusion imaging: a study on cardiovascular event prediction. JACC Cardiovasc Imaging. 2019;13(1):180‐182. 31607660 10.1016/j.jcmg.2019.08.009

[10] Alonso DH , Wernick MN , Yang Y , Germano G , Berman DS , Slomka P . Prediction of cardiac death after adenosine myocardial perfusion SPECT based on machine learning. J Nucl Cardiol. 2019;26(5):1746‐1754. 29542015 10.1007/s12350-018-1250-7 PMC6138585

[11] Benjamins JW , Yeung MW , Maaniitty T , et al. Improving patient identification for advanced cardiac imaging through machine learning‐integration of clinical and coronary CT angiography data. Int J Cardiol. 2021;335:130‐136. 33831505 10.1016/j.ijcard.2021.04.009

[12] Chen T , Guestrin C . XGBoost: A scalable tree boosting system. Proceedings of the 22nd ACM SIGKDD International Conference on Knowledge Discovery and Data Mining. 2016 785–794.

[13] Yeung MW , Benjamins JW , Knol RJJ , et al. Multi‐task deep learning of myocardial blood flow and cardiovascular risk traits from PET myocardial perfusion imaging. J Nucl Cardiol. 2022;29(6):3300‐3310. 35274211 10.1007/s12350-022-02920-x PMC9834343

[14] Juárez‐Orozco LE , van der Zant FM , Slart RHJA , et al. Type 2 diabetes mellitus correlates with systolic function during myocardial stress perfusion scanning with Nitrogen‐13 ammonia PET. J Nucl Cardiol. 2017;25(4):1305‐1311. 10.1007/s12350-016-0482-7 PMC5548822 27083442

[15] Hutchins GD , Schwaiger M , Rosenspire KC , Krivokapich J , Schelbert H , Kuhl DE . Noninvasive quantification of regional blood flow in the human heart using N‐13 ammonia and dynamic positron emission tomographic imaging. J Am Coll Cardiol. 1990;15(5):1032‐1042. 2312957 10.1016/0735-1097(90)90237-j

[16] Cerqueira MD , Weissman NJ , Dilsizian V , et al. Standardized myocardial segmentation and nomenclature for tomographic imaging of the heart. Circulation. 2002;105(4):539‐542. 11815441 10.1161/hc0402.102975

[17] Teuho J , Schultz J , Klén R , et al. Classification of ischemia from myocardial polar maps in ^15^O–H_2_O cardiac perfusion imaging using a convolutional neural network. Sci Rep. 2022;12:2839. 35181681 10.1038/s41598-022-06604-x PMC8857225

[18] Degtiarova G , Garefa C , Boehm R , et al. Radiomics for the detection of diffusely impaired myocardial perfusion: a proof‐of‐concept study using 13 N‐ammonia positron emission tomography. J Nucl Cardiol. 2023;30(4):1474‐1483. 36600174 10.1007/s12350-022-03179-y PMC10371953

[19] Yang J , Guan J . A heart disease prediction model based on feature optimization and smote‐Xgboost algorithm. Information. 2022;13(10):475.

[20] Lehtonen E , Kujala I , Tamminen J , et al. Incremental prognostic value of downstream positron emission tomography perfusion imaging after coronary computed tomography angiography: a study using machine learning. Eur Heart J Cardiovasc Imaging. 2024;25:285‐292. 37774503 10.1093/ehjci/jead246 PMC10824480

[21] Angione C , Silverman E , Yaneske E . Using machine learning as a surrogate model for agent‐based simulations. PLoS One. 2022;17(2):e0263150. 35143521 10.1371/journal.pone.0263150 PMC8830643

