# Supplementary material for: Expanding interpretability through complexity reduction in machine learning‐based modelling of cardiovascular disease: A myocardial perfusion imaging PET/CT prognostic study
Source: Eur J Clin Invest. 2025 Apr 7;55(Suppl 1):e14391. doi: 10.1111/eci.14391 (PMC11973839; doi:10.1111/eci.14391)
Supplement: Supplementary file 1 — Appendix S1. [file ECI-55-e14391-s001.docx]

## Supplementary material: Expanding interpretability through complexity reduction in machine learning -based modelling of cardiovascular disease: A myocardial perfusion imaging PET/CT prognostic study

A short running title of less than 40 characters: Expanding interpretability in a PET/CT study

The full names of all authors, highest academic degrees, and institutional affiliations: Eero Lehtonen^1^ DSc (Tech), Jarmo Teuho^1^ PhD, Monire Vatandoust^1^ MSc, Juhani Knuuti^1^ MD, PhD, Remco J. J. Knol^2^ PhD, Friso M. van der Zant^2^ PhD, Luis Eduardo Juárez-Orozco^1,3^ MD, PhD, and Riku Klén^1^ PhD

Corresponding author: Riku Klén, Turku PET Centre, Turku University Hospital, Kiinamyllynkatu 4-8, FI-20520 Turku, Finland. Telephone +358 2 313 0000; Fax +358 50 574 7933, Email address: riku.klen@utu.fi

^1^Turku PET Centre, Turku University Hospital and University of Turku, 20521 Turku, Finland

^2^Cardiac Imaging Division Alkmaar, Department of Nuclear Medicine, Northwest Clinics, Alkmaar, The Netherlands

^3^Department of Cardiology, Division Heart & Lungs, University Medical Center Utrecht, Utrecht University, Heidelberglaan 100, 3508, GA, Utrecht, The Netherlands

**Acknowledgments.**

Authors acknowledge financial support by grants from the Academy of Finland (PI Dr Juhani Knuuti, Academy Decision Number 351482), the Finnish Cultural Foundation (Maire and Aimo Mäkinen Fund), and the State Research Funding of Turku University Hospital (under grant 30046).

Financial Disclosure. Dr. Knuuti received consultancy fees from GE Healthcare and Synektik and speaker fees from Bayer, Lundbeck, Boehringer-Ingelheim, Pfizer and Siemens, outside of the submitted work. All other authors have reported that they have no relationships relevant to the contents of the paper to disclose.

## Study population

Data from 1185 patients was retrospectively collected and analyzed from the population referred to quantitative PET myocardial perfusion imaging due to suspected myocardial ischemia between 2015 and 2017 at the department of nuclear medicine of the Northwest Clinics, Alkmaar, The Netherlands. Patients with documented CAD either as prior myocardial infarction (MI) or revascularization (percutaneous coronary intervention or coronary artery bypass graft surgery), were excluded from the study. All patients gave written informed consent for use of their anonymized data. Besides standard imaging protocol and clinical management no additional measurements or actions affecting the patient were performed. Approval of the local ethical committee for the present study was not necessary since the study does not fall within the scope of the Dutch Medical Research Involving Human Subjects Act (section 1.b WMO, 26th February 1998). The study population and the data collection protocol have been previously reported in [1].

## PET Data Acquisition and Quantitative Perfusion Analysis

Every patient underwent a two-phase (rest and adenosine stress) PET scan with the use of ^13^N-ammonia as the perfusion radiotracer. All image data were acquired in list mode on a Siemens Biograph-16 TruePoint PET/CT (Siemens Healthcare, Knoxville, USA) with the TrueV option (the axial field of view of 21.6 cm). This 3D system consists of a 16-slice CT and a PET scanner with four rings of lutetium oxyorthosilicate (LSO) detectors. Patients were instructed to fast overnight and to avoid the consumption of methylxanthines for 24 hours before the study. The details of the acquisition-reconstruction protocol have been published previously in [2].

Based on the dynamic subsets, left ventricular contours were assigned automatically using the SyngoMBF software (Siemens Medical Solutions, Berlin, Germany) with minimum observer intervention when appropriate. With a previously described 2-compartment kinetic model for the aforementioned tracer, value of stress MBF, rest MBF, and MFR were computed and color-coded with a standard scale (stress MBF: 0.0 – 2.7 mL/g/min, rest MBF: 0.0 – 1.0 mL/g/min, MFR: 0.0 – 3.0) for each sample on the polar map through the resulting time-activity curves for quantification [3]. The resulting rest, stress, and reserve polar maps were further analyzed through machine learning as specified in the following.

## Preprocessing of the Polar Map Images from DICOM Reports

The polar maps were originally included in a summary report extracted from the SyngoMBF software, contained within a single DICOM secondary capture image with screen size of 1172 x 1200 pixels. A processing pipeline was prepared in Python (version 3.9.7) to extract each polar map as a separate .png file with individual size of 228 x 228 pixels. Cropping boundaries and the cropping quality of each polar map were inspected manually. Automatic detection was performed by comparison if two separate images contained identical pixel values, or if a single image contained only null values. These cases were then manually inspected and removed if duplicate values or images containing no data were found. After the removal of invalid data entries, polar maps from 1079 patients were retained for further analysis.

## Graphical example of using the sum-of-sigmoids model


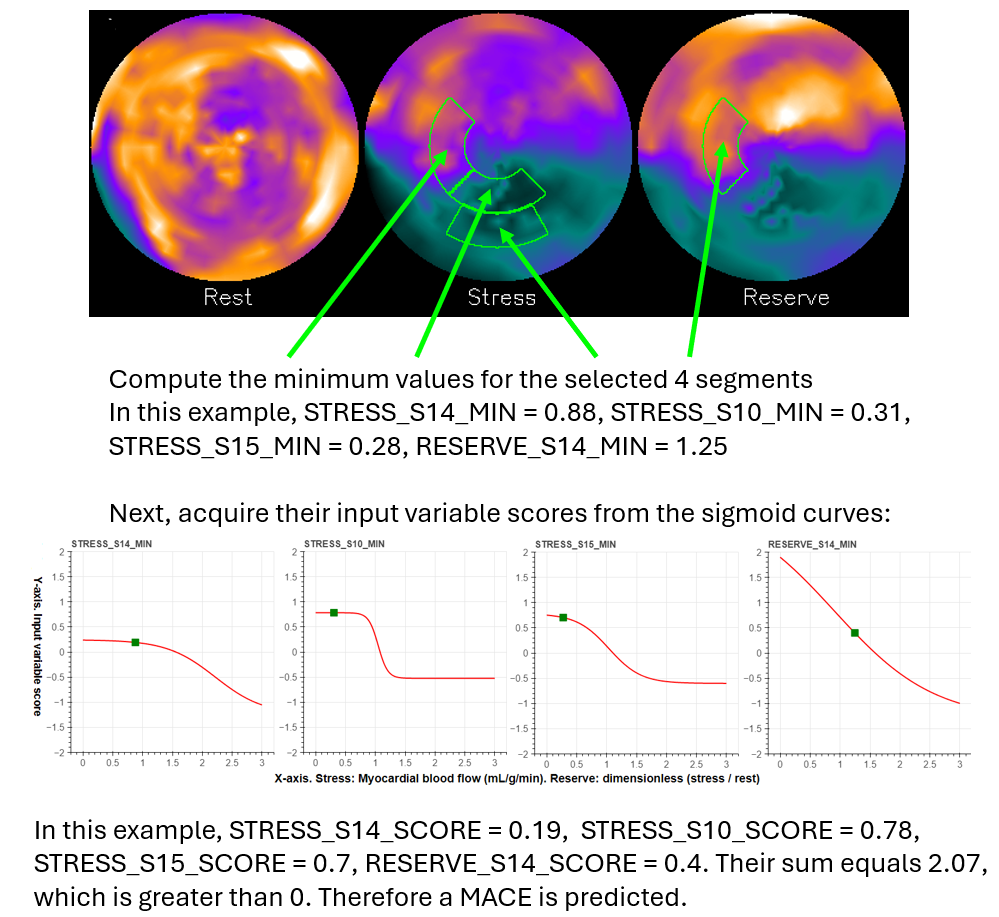


## References

[1] Yeung MW, Benjamins JW, Knol RJJ et al. Multi-task deep learning of myocardial blood flow and cardiovascular risk traits from PET myocardial perfusion imaging. J Nucl Cardiol. 2022;29(6):3300–3310.

[2] Juárez-Orozco LE, van der Zant FM, Slart RHJA, Lazarenko SV, Alexanderson E, Tio RA, Knol RJJ. Type 2 diabetes mellitus correlates with systolic function during myocardial stress perfusion scanning with Nitrogen-13 ammonia PET. J Nucl Cardiol. 2017;25(4):1305–1311.

[3] Hutchins GD, Schwaiger M, Rosenspire KC, Krivokapich J, Schelbert H, Kuhl DE. Noninvasive quantification of regional blood flow in the human heart using N-13 ammonia and dynamic positron emission tomographic imaging. J Am Coll Cardiol. 1990;15(5):1032–1042.
